# Anatomic restoration of an isolated medial tibial plateau depression in a young patient after high-energy trauma: A case report

**DOI:** 10.1097/MD.0000000000050600

**Published:** 2026-09-04

**Authors:** Ahmet Polat, Emin Can Balci

**Affiliations:** a Department of Orthopaedics and Traumatology, Bağcilar Eğitim ve Araştirma Hastanesi, Istanbul, Turkey.

**Keywords:** bone grafting, case report, medial tibial plateau, subchondral fixation, tibial plateau fracture

## Abstract

**Rationale:**

Isolated medial tibial plateau depression fractures are uncommon injuries. Elevation of the depressed articular surface is traditionally supplemented with bone grafting or bone substitute to support the subchondral defect. However, the necessity of graft augmentation in selected fracture patterns remains uncertain.

**Patient concerns:**

An 18-year-old male presented with right knee pain and swelling following a high-energy motorcycle accident.

**Diagnoses:**

Radiographs and computed tomography demonstrated an isolated medial tibial plateau depression fracture with minimal displacement (≤ 10 mm), classified as Arbeitsgemeinschaft für Osteosynthesefragen/Orthopaedic Trauma Association 41-B1.2.

**Interventions:**

The depressed articular surface was anatomically elevated and stabilized using a proximal medial locking plate without bone graft or bone substitute augmentation.

**Outcomes:**

The patient remained non-weight-bearing for 12 weeks, and radiographic union was achieved by 3 months. At the 12-month follow-up, radiographs demonstrated maintained anatomic reduction with < 1 mm residual articular depression, no implant-related or wound complications, and the Knee Injury and Osteoarthritis Outcome Score improved from 2 to 71.

**Lessons:**

This case suggests that, in carefully selected isolated medial tibial plateau depression fractures, stable anatomic reduction and rigid subchondral fixation may provide favorable clinical and radiographic outcomes without routine graft augmentation. Larger clinical studies are needed to further define the indications for this treatment strategy.

## 1. Introduction

Medial tibial plateau fractures frequently occur following high-energy trauma mechanisms. Because the medial compartment is the primary varus load-bearing column of the knee, this region has a distinct biomechanical vulnerability and surgical consequences. This case illustrates that stable anatomic articular restoration and favorable clinical and radiographic outcomes can be achieved in selected isolated medial tibial plateau depression fractures through meticulous reduction and stable fixation.^[1,2]^

## 2. Case presentation

**Figure 1.**
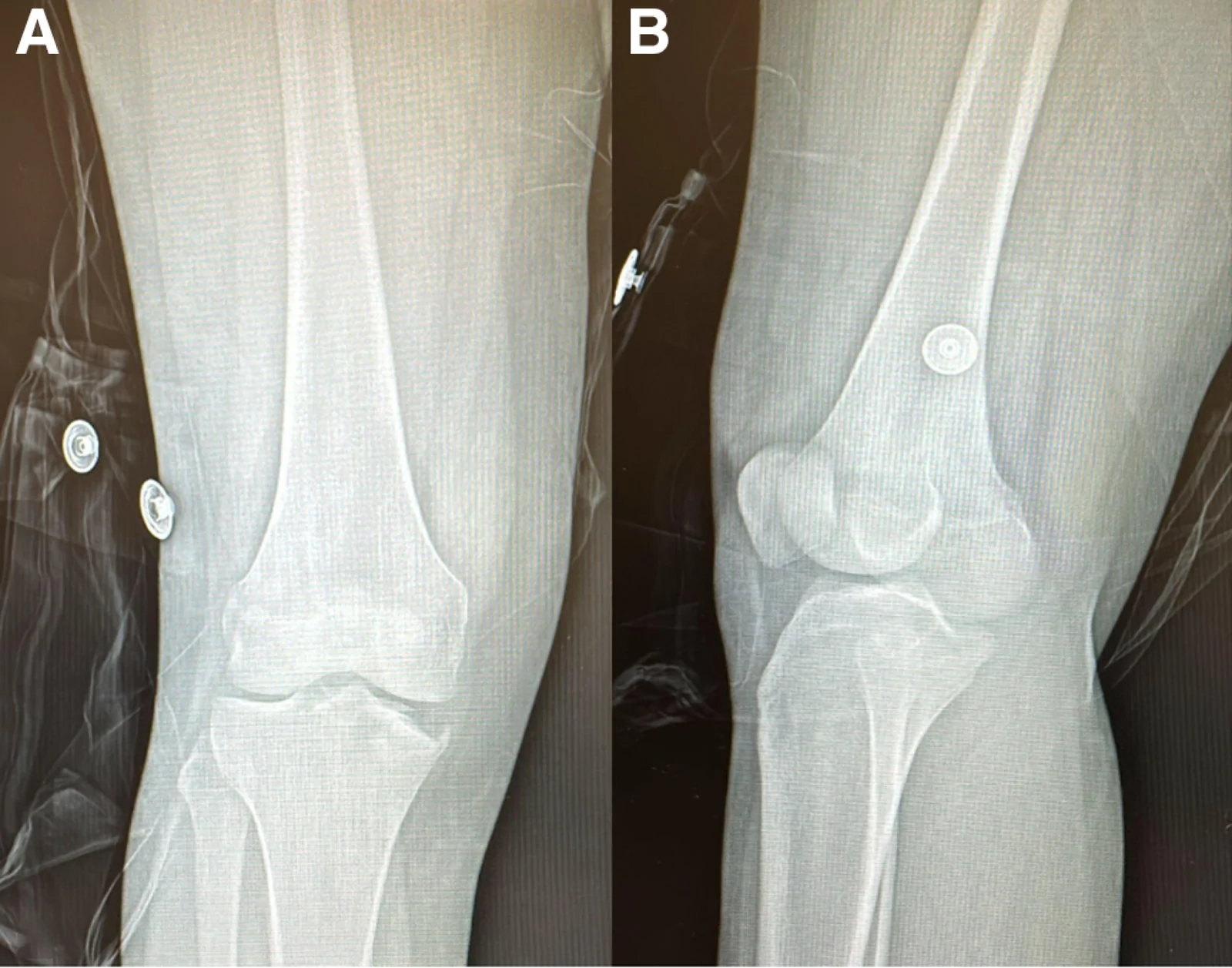
Preoperative radiographs. (A) AP radiograph demonstrating an isolated medial tibial plateau depression fracture; (B) lateral radiograph demonstrating depression of the medial tibial plateau without significant posterior displacement. AP = anteroposterior.

**Figure 2.**
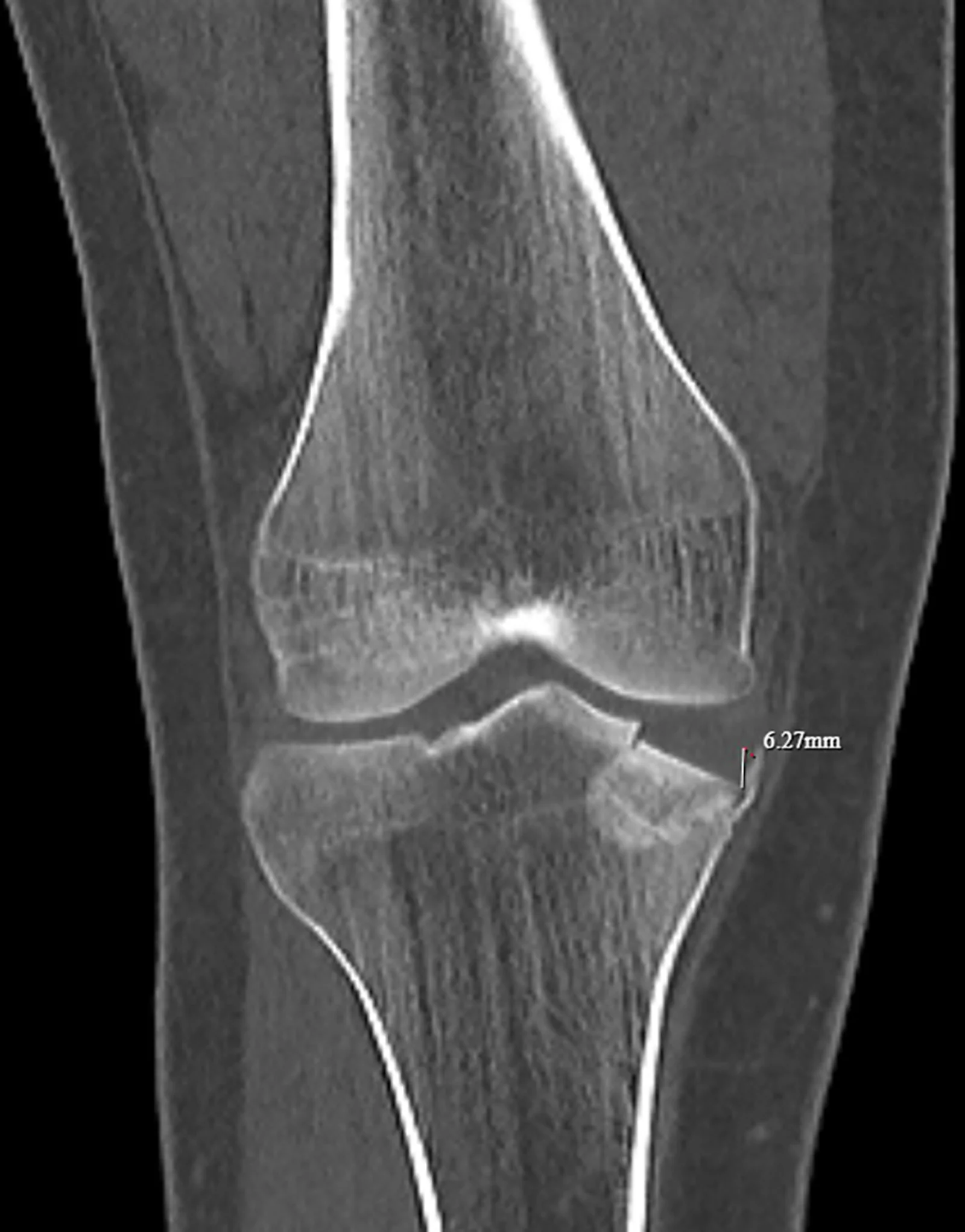
Preoperative CT. Coronal CT image confirming an isolated medial tibial plateau depression fracture with minimal displacement (≤ 10 mm), allowing accurate assessment of the articular depression and preoperative planning. CT = computed tomography.

The patient was an 18-year-old male motorcycle rider who collided laterally with another vehicle and landed directly on his right knee. Physical examination revealed medial swelling and ecchymosis, approximately 20° loss of knee flexion, stable varus–valgus stress (both at 30° flexion and full extension), and intact distal neurovascular status. Preoperative radiographs demonstrated pure medial depression (Figs. 1A and 1B). Computed tomography demonstrated isolated medial plateau depression with minimal displacement (≤ 10 mm) (Fig. 2A). The fracture was classified Arbeitsgemeinschaft für Osteosynthesefragen/Orthopaedic Trauma Association 41-B1.2.

**Figure 3.**
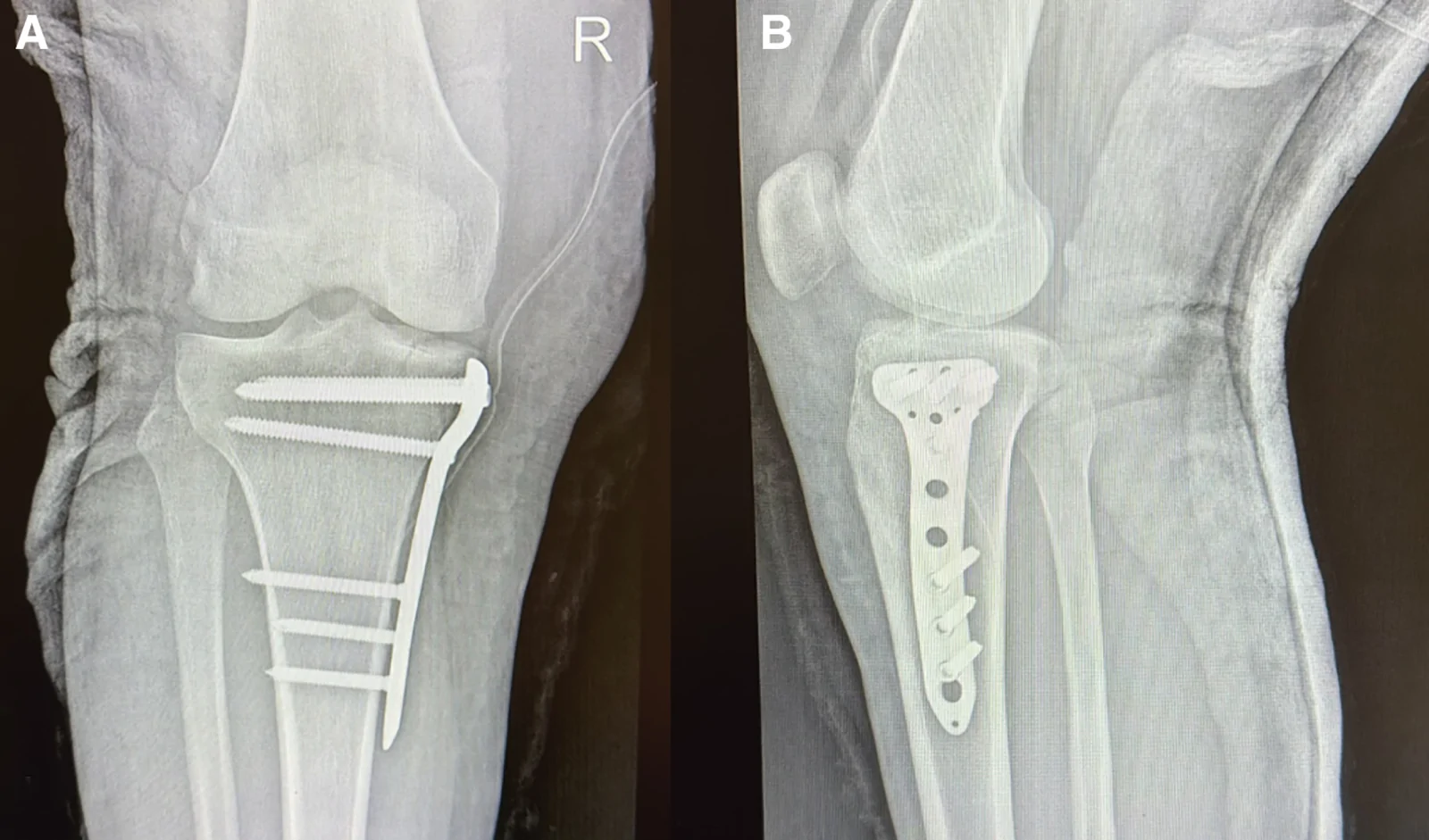
Immediate postoperative radiographs. (A) AP radiograph demonstrating anatomic reduction of the articular surface and stable fixation using a proximal medial locking plate; (B) lateral radiograph confirming restoration of the joint surface and stable subchondral support without residual displacement. AP = anteroposterior.

Surgery was performed on day 4 post-injury with tourniquet use. A standard anteromedial approach was used. The depressed articular surface was elevated under fluoroscopy. No graft or bone substitute was used. A proximal tibial anatomic locking plate was applied, and stable fixation was confirmed intraoperatively (Figs. 3A and 3B).

**Figure 4.**
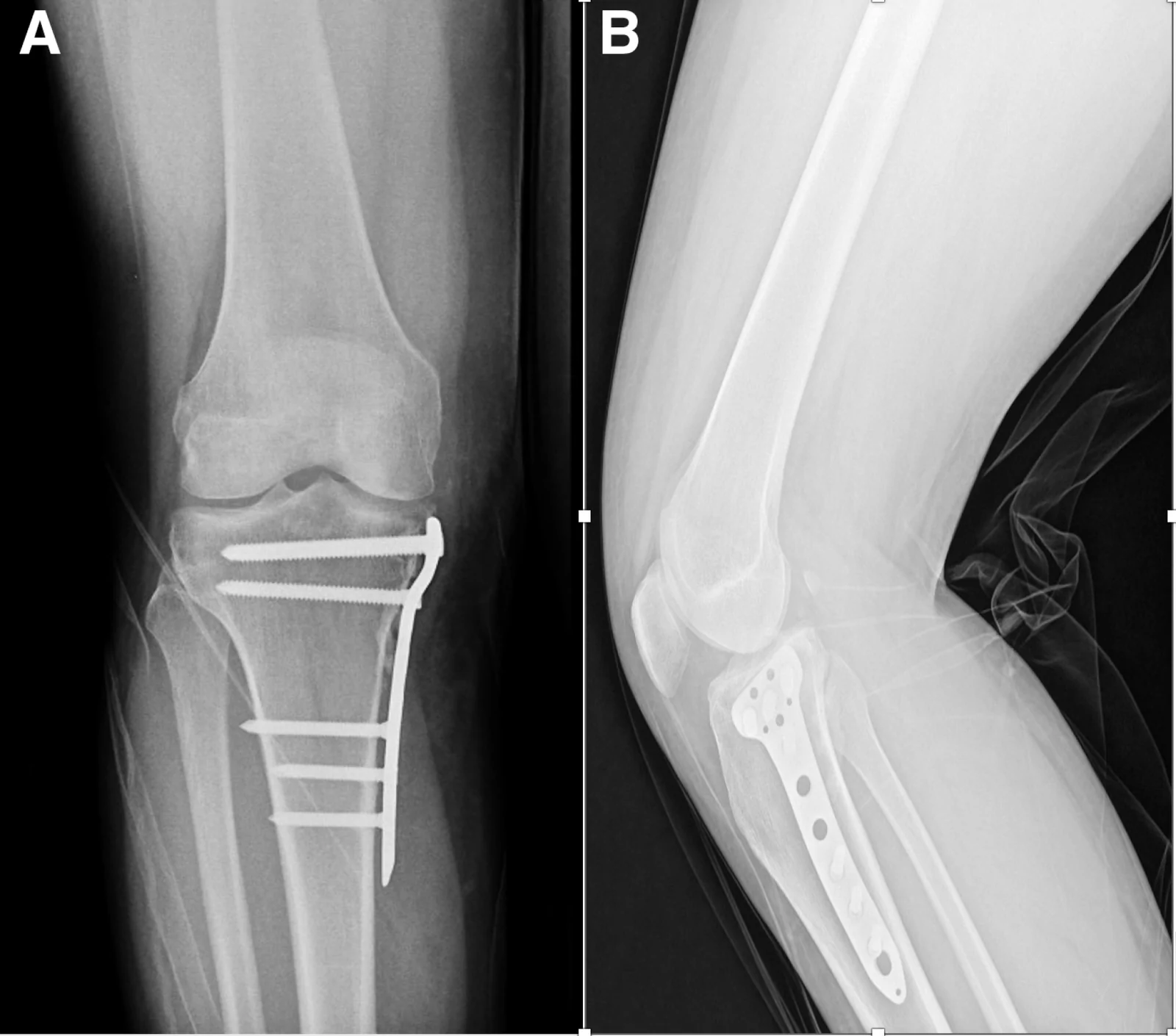
Twelve-month postoperative radiographs. (A) AP radiograph demonstrating complete fracture union, maintained anatomic reduction, and stable implant position; (B) lateral radiograph demonstrating preservation of articular congruity without secondary collapse, loss of reduction, or implant-related complications. AP = anteroposterior.

Postoperatively the limb was splinted until suture removal; then no brace was used. The patient remained non-weight-bearing for 12 weeks, followed by progressive weight-bearing and supervised rehabilitation. Fracture union was achieved at 3 months. At the 12-month follow-up, radiographs demonstrated maintained anatomic reduction without secondary loss of reduction or implant failure (Figs. 4A and 4B). Residual articular depression remained < 1 mm, and no wound- or implant-related complications were observed. The Knee Injury and Osteoarthritis Outcome Score improved from 2 preoperatively to 71 at the final follow-up. The Rasmussen radiographic score at the final follow-up was 18.

## 3. Discussion

Medial tibial plateau depression injuries are mechanically unique because varus load transmission makes subchondral support critical.^[3–5]^ Subchondral rafting and anatomic medial plate constructs are foundational principles in restoring articular congruity in this region.^[1,2]^ In this case, computed tomography-guided preoperative planning, meticulous anatomic elevation, redistribution of local metaphyseal cancellous bone into the metaphyseal defect, and stable fixation with subchondral rafting screws and a medial locking plate maintained the restored joint line without the need for additional graft augmentation.

Although bone grafting or bone substitutes are traditionally recommended to support elevated articular fragments and metaphyseal defects, the present case suggests that additional graft augmentation may not be mandatory in carefully selected fracture patterns when the metaphyseal defect is limited, local cancellous bone can be redistributed into the defect, and stable subchondral fixation is achieved.^[6,7]^ Nevertheless, this observation should not be generalized to all tibial plateau fractures, particularly those with extensive metaphyseal bone loss or severe comminution.^[6]^

Further clinical studies with larger patient cohorts, longer follow-up, and comparative evaluation of grafted and non-grafted techniques are required to better define the indications for graft augmentation in isolated medial tibial plateau depression fractures.

## 4. Conclusion

In selected isolated medial tibial plateau depression fractures, meticulous anatomic reduction and stable fixation with a medial locking plate may maintain anatomic reduction and provide favorable clinical and radiographic outcomes without additional graft augmentation. Further studies are required to define the indications for this graft-sparing approach.

## Author contributions

**Data curation:** Ahmet Polat.

**Investigation:** Ahmet Polat, Emin Can Balci.

**Project administration:** Ahmet Polat.

**Writing** – **original draft:** Ahmet Polat.

**Writing** – **review & editing:** Ahmet Polat, Emin Can Balci.

**Resources:** Emin Can Balci.
